# AMPA receptor auxiliary subunits emerged during early vertebrate evolution by neo/subfunctionalization of unrelated proteins

**DOI:** 10.1098/rsob.200234

**Published:** 2020-10-28

**Authors:** David Ramos-Vicente, Àlex Bayés

**Affiliations:** 1 Molecular Physiology of the Synapse Laboratory, Biomedical Research Institute Sant Pau, Barcelona, Spain; 2 Universitat Autònoma de Barcelona, Barcelona, Spain

**Keywords:** AMPA receptor auxiliary subunit, TARP, cornichon, shisa, Dispanin C, phylogeny

## Abstract

In mammalian synapses, the function of ionotropic glutamate receptors is critically modulated by auxiliary subunits. Most of these specifically regulate the synaptic localization and electrophysiological properties of AMPA-type glutamate receptors (AMPARs). Here, we comprehensively investigated the animal evolution of the protein families that contain AMPAR auxiliary subunits (ARASs). We observed that, on average, vertebrates have four times more ARASs than other animal species. We also demonstrated that ARASs belong to four unrelated protein families: CACNG-GSG1, cornichon, shisa and Dispanin C. Our study demonstrates that, despite the ancient origin of these four protein families, the majority of ARASs emerged during vertebrate evolution by independent but convergent processes of neo/subfunctionalization that resulted in the multiple ARASs found in present vertebrate genomes. Importantly, although AMPARs appeared and diversified in the ancestor of bilateral animals, the ARAS expansion did not occur until much later, in early vertebrate evolution. We propose that the surge in ARASs and consequent increase in AMPAR functionalities, contributed to the increased complexity of vertebrate brains and cognitive functions.

## 1. Introduction

Ionotropic glutamate receptors are key to the physiology of the nervous system, as they mediate fast excitatory neurotransmission [1]. Previously, we reported that the evolution of the proteins that form these tetrameric receptors has been much more sophisticated than previously acknowledged [2]. We found that beyond the well-known AMPA, Kainate, NMDA and Delta classes, there are four other classes exclusive to invertebrate species. Because of this, species with simple nervous systems, such as the sea anemone *N. vectensis*, have a similar number of ionotropic glutamate receptor subunits to animals with complex brains, such as mammals [2]. We argued that the high diversity of neuronal types [3] or the wide array of glutamate receptor functionalities [4] found in vertebrates is unlikely to be the result of an increased repertoire of genes coding for them. Notably, the subcellular traffic and function of ionotropic glutamate receptors is controlled by their auxiliary subunits [5]. These proteins add a new layer of complexity to glutamatergic transmission and might have contributed to an expanded functionality of these receptors in animals with complex brains. In mammals, sixteen proteins have been identified as ionotropic glutamate receptor auxiliary subunits. Of these, fourteen modulate AMPA-type glutamate receptors (AMPARs) [6], and they are referred to as AMPA receptor auxiliary subunits (ARASs). Only one auxiliary subunit has been reported for NMDA receptors, Neto1 [7], which also regulates Kainate receptors, as does Neto2 [8,9]. Currently known mammalian ARASs belong to five protein families: CACNG and GSG1 (both within the superfamily of claudins), cornichon, shisa and Dispanin C.

Genes belonging to the same family are defined as paralogues; these originate from gene or genome duplication events [10]. In vertebrates, many paralogues resulted from the two rounds of whole-genome duplication (2R) occurred at the base of this lineage, approximately 400 Ma [11]. After duplication, the two new genes can experience processes of neofunctionalization or subfunctionalization. In the first scenario, one of the new genes retains all functions performed by the ancestral gene, while the second acquires new ones [12,13]. Alternatively, genes subfunctionalize when the multiple functions carried out by the ancestral gene are realized separately by descendent paralogues [13,14]. Establishing if paralogues have undergone a process of neo or subfunctionalization requires a precise understanding of all functions carried out by the ancestral gene and the descending paralogues. When this knowledge is not available, it is not possible to determine which one of these two processes took place [15]; in these occasions, the term neo/subfunctionalization can be used [13].

Known invertebrate ARAS belong to the CACNG or cornichon families. These have been described in *C. elegans* [16,17] and the fruit fly [18,19]. The first ARAS to be reported was CACNG2, also called stargazin, as it is mutated in *stargazer* mice [20]. Although this protein is phylogenetically related to CACNG1, an auxiliary subunit of voltage-dependent calcium channels (VDCC), it specifically interacts with AMPAR [21,22]. Subsequently, six other CACNGs have been discovered in mammals (CACNG3 to CACNG8 [23,24]). Apart from CACNG6, which also acts as a VDCC auxiliary subunit [25,26], all other CACNGs function as AMPA receptor auxiliary subunits [27–29]; these are usually referred to as TARPs (transmembrane AMPA receptor regulatory proteins) [27]. Phylogenetic analysis of mammalian TARPs identifies that they are more related to each other than to auxiliary subunits of VDCCs [25,27], being divided in two types [27,30,31] that differentially modulate AMPAR [28,29]. The most recently identified ARAS is GSG1L [32,33]. In vertebrate genomes, GSG1L has two paralogues: GSG1, which interacts with the polymerase TPAP, and GSG1L2 [32,34], of unknown biological function. These proteins also belong to the superfamily of claudins, like CACNGs. Despite its structural similarity with TARPs, GSG1L modulates AMPAR in a different way, downregulating its traffic to the plasma membrane and accelerating deactivation and desensitization [35,36]. The remaining ARASs have been identified in three other protein families: cornichon, shisa and Dispanin C. In all cases, these families contain ARAS and proteins with different biological functions. Among cornichons, CNIH2 and CNIH3 modulate AMPAR function, but CNIH1 and CNIH4 regulate the traffic of TGF*α* and GPCRs, respectively [37–41]. Four members of the shisa family, Shisa6 to Shisa9 (originally referred to as CKAMPs [42–46]), interact with AMPAR, yet Shisa2 and Shisa3 are involved in the traffic of FGF [47] and WNT [48] receptors, respectively, and Shisa5 participates in the p53/TP53-dependent apoptosis pathway [49]. Finally, SynDIG1, from the Dispanin C family, has also been identified as an ARAS [50,51], this protein has two paralogues in vertebrates, SynDIG1L and TMEM91, with poorly understood functions.

The evolutionary origin of ARASs is well established for cornichons, which have been identified in the ancestor of eukaryotes and are present in a large range of species, including plants and yeasts [52,53]. The claudin superfamily, to which CACNG and GSG1 proteins belong, present homologues in the basal metazoan phylum of porifera [54,55], placing its origin prior to the divergence of these organisms. While CACNG homologues have been reported in different bilaterian species, including vertebrates and the fruit fly or *C. elegans* [17,19], it is still unknown when GSG1s appeared during evolution. In addition, some shisa homologues have been described in porifera and Dispanin C homologues have been identified in brown algae [56,57]. Here, we present a comprehensive study of the animal evolution of all protein families that include AMPAR auxiliary subunits. Our work shows that cornichons, present in the ancestor of all metazoans, would be the most ancient of all ARAS. TARPs, which appeared together with AMPARs in the ancestor of bilaterians, also function as ARASs in invertebrate organisms. Nevertheless, most ARASs, including shisas, GSG1L and SynDIG1, would have been recruited to modulate AMPAR function early in vertebrate evolution, suggesting that during this period there was an evolutionary pressure that favoured an expansion of the functionality of AMPARs.

## 2. Materials and methods

### 2.1. Identification of genes coding for AMPA receptor auxiliary subunits in metazoan genomes

Phylogenetic analysis was performed as described previously [2]. We searched sequences in 31 species belonging to different metazoan phyla: Porifera, Ctenophora, Placozoa, Cnidaria, Lophotrochozoa, Ecdysozoa, Hemichordata, Chordata and Vertebrata. The same species were used to construct all phylogenies. We used slowly evolving species whenever possible. All sequences were retrieved from public databases. *A. digitifera* and *P. flava* sequences were obtained from the Marine Genomics Unit [58,59]. A complete list of species included in the analysis and the corresponding database where sequence search was done can be found in electronic supplementary material, table S1.

Sequences from protein families of interest were identified by homology-based searches with reciprocal identifications. Mouse proteins were used as search queries, when different isoforms were present we used the longest amino acid sequence. We searched for homologues using the BLASTP and TBLASTN tools [60] with default parameters. Subject sequences with an E-value below 0.05 were selected as candidate homologues. These sequences were re-blasted against mammal proteins in the NCBI database of ‘non-redundant protein sequences' using the BLASTP and BLASTX tools, respectively. Identified homologues which have a protein length that is less than 50% of the query protein were discarded.

### 2.2. Phylogenetic analyses

The CACNG-GSG1 tree was constructed using a total of 114 sequences, from which fifteen are used as the outgroup (electronic supplementary material, file S1). The cornichon tree includes 70 sequences, of which five are cornichons from *Arabidopsis thaliana* used as the outgroup (electronic supplementary material, file S2). The shisa tree includes 98 sequences, eight of which are used as the outgroup (electronic supplementary material, file S3). Finally, the Dispanin C tree includes 41 sequences, four of which were used as the outgroup (electronic supplementary material, file S4).

Protein sequences were aligned with the MUSCLE algorithm [61], included in the software package MEGAX [62] with default parameters. ProtTest v3.4.2 was used to establish the best evolutionary model [63]. Trees were constructed using MrBayes v3.2.7 [64] for Bayesian inference and IQ-TREE [65] for maximum-likelihood analysis. For Bayesian inference phylogenies were run for 10 000 000 generations. Markov chain Monte Carlo (MCMC) was used to approximate the posterior probability of the Bayesian trees. Bayesian analyses included two independent MCMC runs, each using four parallel chains composed of three heated and one cold chain. Twenty-five per cent of initial trees were discarded as burn-in. Convergence was assessed when the potential scale reduction factor (PSRF) value was between 1.002 and 1.000. In maximum-likelihood analysis the starting tree was estimated using a neighbour-joining method and branch support was obtained after 1000 iterations of ultrafast bootstrapping [66]. Gene/protein names were given based on their position in the tree. Phylogenetic trees were rendered using FigTree (http://tree.bio.ed.ac.uk/software/figtree/). Phylogenetic calculations were performed at the CIPRES science gateway [67].

### 2.3. Protein nomenclature

Proteins from non-vertebrate species were systematically named following this nomenclature: (i) the name of the subfamily which they belong, or family if the sequence is not assigned to a subfamily; (ii) a Greek letter to identify non-vertebrate paralogues, if any; and (iii) a three-letter species code.

### 2.4. Prediction of PDZ motifs classes

The software Eukaryotic Linear Motif (ELM) [68] was used to identify if the C-terminus of proteins presented a PDZ binding motif and, if so, to what class it belonged.

## 3. Results

### 3.1. TARPs are ancient AMPAR auxiliary subunits widespread in bilaterians

**Figure 1.**
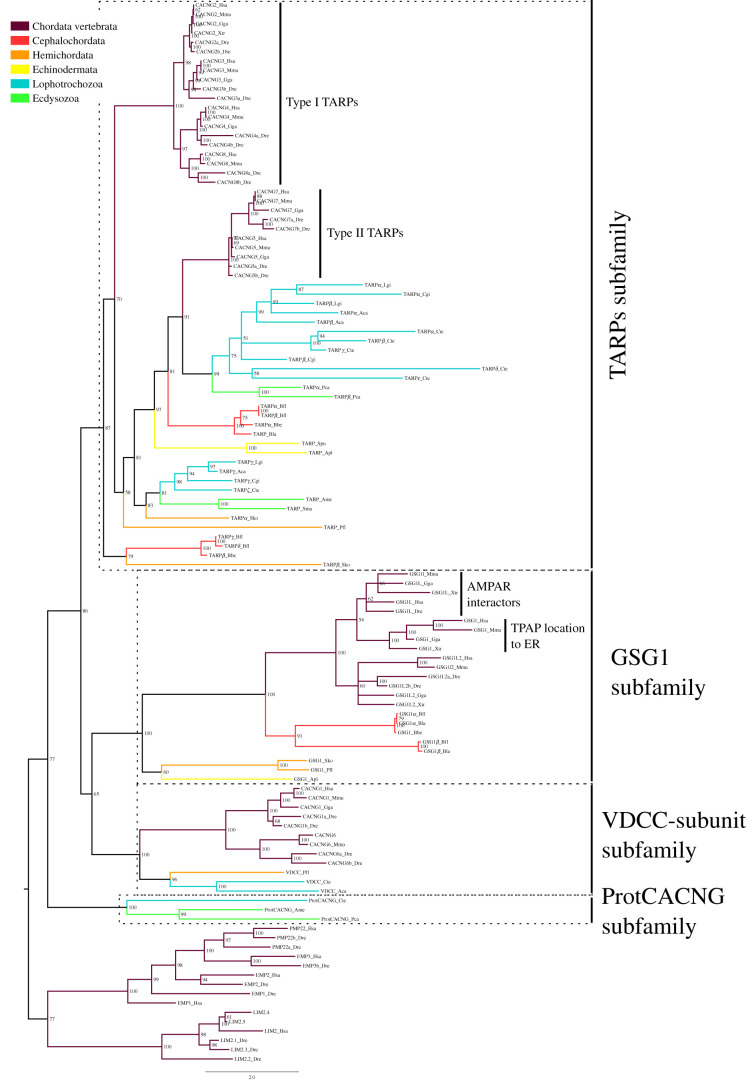
Bayesian inference phylogenetic tree of the CACNG-GSG1 family. The subfamilies in which the CACNG-GSG1 protein family is divided are highlighted by dashed line boxes. The name of each subfamily is presented at the right of the corresponding box. Posterior probabilities are shown at tree nodes and protein names at the end of branches. Tree branches are coloured based on phylum, as indicated in the legend. The closest relatives to vertebrate CACNG in the claudin superfamily were used as outgroup. The Type I and Type II TARPs are indicated. If known, the function of vertebrate GSG1 subfamily sequences was also included. Scale bar denotes number of amino acid substitutions per site. The amino acid substitution model used was Vt + G + F, the analysis ran for 10 000 000 generations, the final standard deviation was 0.012330 and the final potential scale reduction factor (PSRF) was 1.000.

Among protein families including AMPA receptor auxiliary subunits, CACNGs and GSG1s belong to the superfamily of claudins [32,69,70]. We thus incorporated GSG1s into the phylogeny of CACNGs to investigate if they are part of the same family within the claudin superfamily. We found that GSG1 proteins confidently fell within the ingroup (figure 1; electronic supplementary material, figure S1). Thus CACNGs and GSG1s are more evolutionarily related to each other than they are to the rest of the claudin superfamily, belonging to the same family, which we have named CACNG-GSG1. We only found CACNG-GSG1 sequences in bilaterian species (table 1), indicating that this family appeared in a common ancestor of bilaterians. Phylogenetic analysis identified four subfamilies among CACNG-GSG1s: TARPs, GSG1s, VDCCs and a protostome-specific subfamily. As these are widely represented among bilaterian phyla, we propose that the ancestral CACNG-GSG1 experienced three duplication events before the split of bilaterians (electronic supplementary material, figure S2A). Nevertheless, only the TARP subfamily is conserved in all bilaterians investigated. The other families were lost in certain lineages. For instance: (i) the GSG1 subfamily is only present in deuterostomes; (ii) molluscs would have lost the protostome-specific subfamily; and (iii) the VDCC-subunit subfamily was independently lost by cephalochordates, echinodermates and ecdysozoans.

**Table 1.** Number of proteins found in the four families known to include AMPA receptor auxiliary subunits (ARASs). The number of ARASs is given in brackets. Proteins from *Homo sapiens* are experimentally confirmed as ARAS. Invertebrate proteins are defined as candidate ARASs based exclusively in the phylogenies.

		phylum	species	CACNG-GSG1	CNIH	Shisa	Dispanin C
Metazoa	Bilateria	Vertebrata	*H. sapiens*	11 (7)	4 (2)	10 (4)	3 (1)
		Chordata	*C. intestinalis*	0	2 (1)	0	0
			*B. lanceolatum*	3 (1)	3 (1)	6 (0)	1 (1)
			*B. belcheri*	3 (2)	2 (1)	6 (0)	7 (1)
			*B. floridae*	6 (4)	3 (2)	8 (0)	3 (0)
		Hemichordata	*S. kowalevskii*	3 (2)	2 (1)	16 (0)	1 (1)
			*P. flava*	3 (1)	1 (0)	4 (0)	0
		Echinodermata	*A. planci*	2 (1)	1 (1)	2 (0)	0
			*S. purpuratus*	1 (1)	1 (1)	1 (0)	2 (0)
		Mollusca	*L. gigantea*	3 (3)	2 (1)	0	2 (0)
			*C. gigas*	3 (3)	3 (1)	0	1 (1)
			*A. californica*	4 (3)	3 (2)	1 (0)	0
		Annellida	*C. teleta*	8 (6)	2 (1)	2 (0)	1 (0)
		Arthropoda	*A. mellifera*	2 (1)	1 (1)	0	0
			*S. maritima*	1 (1)	1 (0)	1 (0)	0
		Priapullida	*P. caudatus*	3 (2)	2 (1)	0	0
	non-Bilateria	Cnidaria	*N. vectensis*	0	2 (1)	0	0
			*A. digitifera*	0	2 (1)	0	0
			*O. faveolata*	0	2 (1)	0	0
			*H. magnipapillata*	0	1 (1)	0	1 (0)
		Placozoa	*T. adhaerens*	0	2 (1)	0	0
		Porifera	*O. carmela*	0	2 (1)	0	2 (1)
			*S. cilliatum*	0	1 (0)	0	0
			*L. complicata*	0	2 (1)	0	1 (0)
			*A. queenslandica*	0	2 (1)	0	0
		Ctenophora	*M. leidyi*	0	1 (1)	0	1 (0)

Among the phyla studied, vertebrates present the highest number of CACNG-GSG1s (11 proteins; table 1), of which 7 are ARAS. Although *Capitella teleta*, an annelid, has eight members in this family, of which six would be TARPs according to the phylogenies, most invertebrates investigated generally have fewer CACNG-GSG1s. All mammalian proteins within the branch of TARPs are known AMPAR regulatory proteins [27,28,71]. Due to their position in the trees we propose that invertebrate orthologues to vertebrate TARPs and their bilaterian ancestor would interact and regulate AMPARs. However, with the exception of cephalochordate sequences, invertebrate TARPs are more divergent than their vertebrate counterparts, as indicated by their longer branches. It is thus plausible that the ability to interact with AMPARs has been altered in this species. Functionally, vertebrate TARPs are classified into Type I and Type II [27,28,71]. This classification is mirrored by the phylogenies, as vertebrate sequences form two monophyletic groups, one for each type. While invertebrate proteins cannot be unambiguously related to Type I or Type II TARPs, as the statistics metrics generated by the Bayesian (figure 1) and maximum-likelihood (electronic supplementary material, figure S1) phylogenies are not high enough, the trees suggest that the majority of them belong to Type II and that Type I would have been lost in non-vertebrates. Among GSG1s, vertebrate GSG1Ls are the only ones known to act as ARASs [32,33]. Yet, our tree indicates that all GSG1 paralogues arouse from recent duplications at the base of the vertebrate phylum, suggesting that the emergence of ARAS in this family occurred by neo/subfunctionalization early in vertebrate evolution.

### 3.2. Sequence analysis reveals common motifs in CACNG-GSG1s

**Figure 2.**
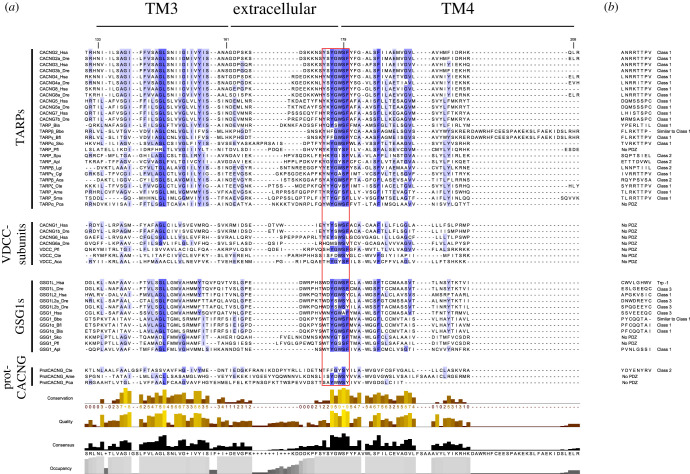
Multiple sequence alignment of the TM3, TM4 and PDZ binding motif of CACNG-GSG1 proteins. The sequence alignment includes sequences from the four subfamilies in the CACNG-GSG1 protein family. From vertebrate species, only *Homo sapiens* and *Danio rerio* sequences were included. In the TARPs subfamily, only representative sequences from invertebrate species are shown. A complete alignment of TARPs sequences can be seen in electronic supplementary material, figure S3. The name of the subfamilies is indicated at the left of sequence names. (*a*) Alignment of the TM3 and TM4 of CACNG-GSG1s proteins, which are implicated in the interaction with AMPAR. The extension of the transmembrane segments and the extracellular loop is marked on the top of the alignment. Residues involved in the highly conserved YSYGWSF motif are highlighted by a red box. The conservation of each position of the alignment is represented by an intensity gradient of the background, higher conservation corresponding to more intense blue, and by a bar chart at the bottom. Also the quality chart (a measure of the probability of seeing mutation in an alignment position), the consensus sequence and the occupancy chart are shown in the bottom. Residue numbers shown on top indicate the start and finish of each transmembrane helix. Protein numbering corresponds to human CACNG2 sequence. (*b*) The last eight residues of proteins containing a PDZ binding motif are shown. If no PDZ binding motif is found it is labelled as No PDZ. The class of each PDZ binding motif is also displayed. The figure was prepared with Jalview v2.11.0.

We next constructed multiple sequence alignments to investigate primary sequence features among CACNG-GSG1s (figure 2*a*; electronic supplementary material, figure S3). We found a highly conserved motif of 7 residues in all CACNG-SGS1s (consensus sequence: Y(174)SYGWSF, residue numbering corresponds to human CACNG2). The last five residues of this motif present the highest conservation, S179 being the most conserved position. We also investigated which proteins from this family would present PDZ binding motifs, as mammalian TARPs [5] present Class 1 PDZ motifs. These short C-terminal sequences contribute to the anchoring of TARPs at the postsynaptic membrane [72,73]. All TARP subfamily sequences except two, TARP_Pfl and TARP*α*_Pca, are predicted to have a PDZ binding motif; most of these being Class 1 motifs. TARP_Spu, TARP_Apl, TARP*β*_Lgi and TRAP*β*_Aca are predicted to have changed this into Class 2 motifs. Similarly, most GSG1 subfamily proteins also have a PDZ binding motif, except GSG1s from hemichordates (figure 2*b*). These motifs are present in GSG1 proteins that act as ARAS but also in those with other functions. Interestingly, this subfamily presents three types of PDZ motifs. Vertebrate GSG1s are predicted to have Class 3 motifs, although their cephalochordate orthologues would have Class 1 motifs. Mammalian GSG1L2 would also present Class 1 motifs although *Danio rerio* orthologues present a Class 3 motif. Finally, mammalian GSG1Ls, which are the only members of the family known to interact with AMPARs, present a PDZ motif class termed Trp-1 [74]. This motif results from an insertion of 7 amino acids exclusive to the mammalian lineage. Other vertebrate species present a Class 3 PDZ motif (electronic supplementary material, figure S4).

### 3.3. A process of subfunctionalization would have led to vertebrate cornichon ARAS

**Figure 3.**
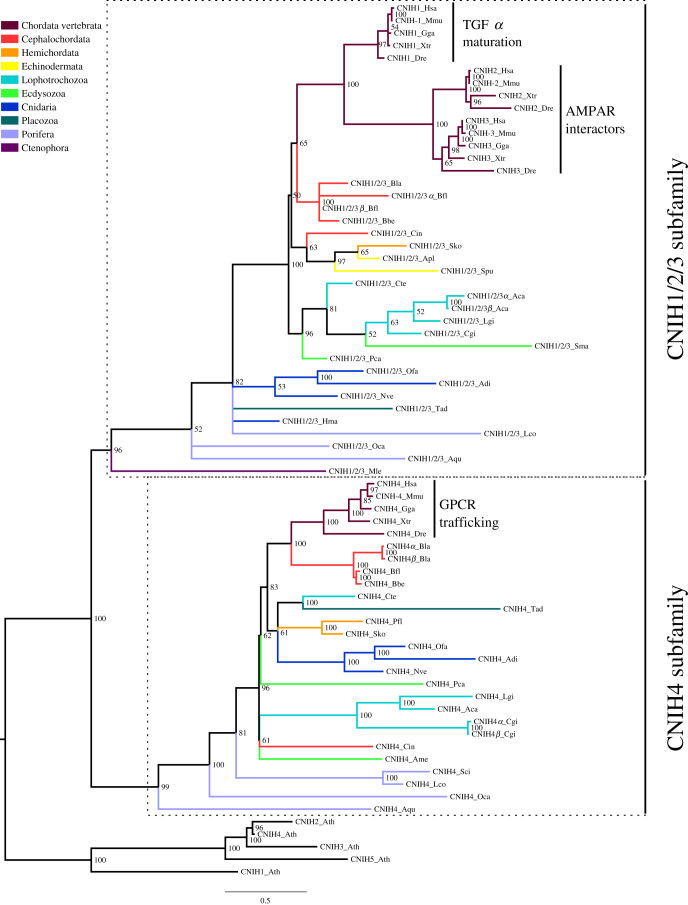
Bayesian inference phylogenetic tree of the cornichon family. The two subfamilies in which the cornichon family is divided are highlighted by dashed line boxes. The name of each subfamily is presented at the right of the box. Posterior probabilities are shown at tree nodes and protein names at the end of each branch. Tree branches are coloured based on phylum, as indicated in the legend. Cornichon proteins from *Arabidopsis thaliana* were used as outgroup. When known the function of vertebrate sequences is indicated. Scale bar denotes number of amino acid substitutions per site. The amino acid substitution model used was Lg + G, the analysis ran for 10 000 000 generations, the final standard deviation was 0.004018 and the final potential scale reduction factor (PSRF) was 1.000.

We next investigated the phylogeny of cornichon proteins. Noticeably, we found cornichon sequences in all species investigated, covering all metazoan phyla (table 1). Our phylogenetic analysis indicates that this family is divided into two subfamilies: CNIH1/2/3 and CNIH4 (figure 3; electronic supplementary material, figure S5). Furthermore, it also shows that the ancestor of all metazoans already presented the two genes that later gave rise to these two subfamilies (electronic supplementary material, figure S2B). Interestingly, both cornichon subfamilies are highly conserved, having experienced few duplication or deletion events throughout metazoan evolution. Nevertheless, while the CNIH1/2/3 subfamily is found in all metazoan phyla studied, ctenophores and echinoderms apparently lost the CNIH4 subfamily. From all species studied, vertebrates have the highest number of cornichon proteins, presenting four. Among invertebrates, *B. lanceolatum*, *B. floridae*, *C. gigas* and *A. californica* are the species with more cornichons, with three coding genes in their genomes.

The vertebrate CNIH4 protein interacts with GPCRs, promoting their traffic to the cell surface [41]. The high conservation of this subfamily allows us to hypothesize that all its members will act in GPCR trafficking to the cell surface, like their vertebrate counterparts. Instead, vertebrate members of the CNIH1/2/3 subfamily have different functions. While CNIH1 is involved in the maturation of TGF*α* [18], both CNIH2 and CNIH3 function as ARASs [33]. These three genes appeared by duplication in the vertebrate ancestor; thus, the phylogeny alone does not allow us to hypothesize about the function of the ancestral gene. Yet both the fruit fly and *C. elegans* present a single cornichon orthologue each [16,18]. Interestingly, the fly protein has been found to participate in the maturation of TGF*α* [18], as mammalian CNIH1s, while the *C. elegans* orthologue, cni-1, acts as an ARAS. To the best of our knowledge the role of the fly protein as ARAS has not been investigated, nor has the role of cni-1 been studied in the context of TFG*α* maturation. Nevertheless, we propose that the invertebrate orthologue of CNIH1, 2 and 3 performs both functions and that in the vertebrate lineage this gene was duplicated and subfunctionalized so that CNIH1 retained the role as a factor for TFG*α* maturation, and the ancestor of CNIH2 and 3 retained the ARAS function.

### 3.4. The phylogeny of the shisa family shows independent expansions in deuterostome species

**Figure 4.**
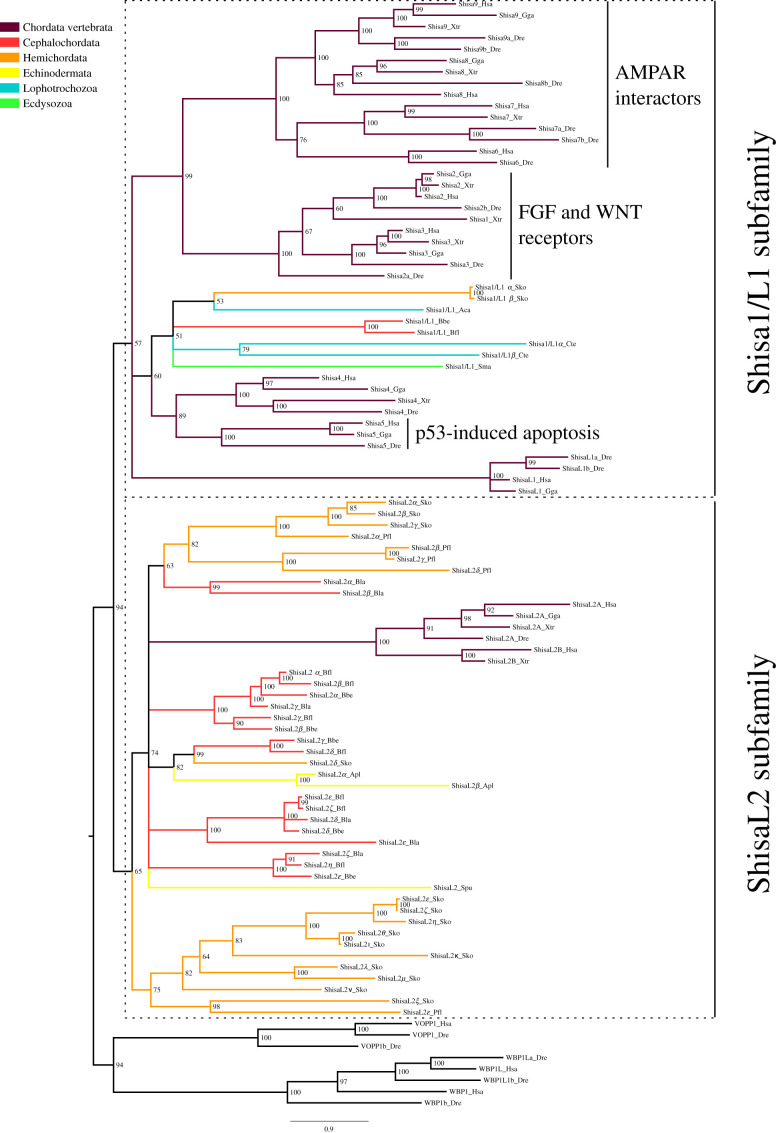
Bayesian inference phylogenetic tree of the shisa family. The two subfamilies in which the shisa family is divided are highlighted by dashed line boxes. The name of each subfamily is presented at the right of the box. Posterior probabilities are shown at tree nodes and protein names at the end of each branch. Tree branches are coloured based on phylum, as indicated in the legend. The closely related vertebrate proteins VOPP and WBP1 were used as outgroup. The function of vertebrate sequences, if known, is indicated. Scale bar denotes number of amino acid substitutions per site. The amino acid substitution model used was Vt + I + G + F, the analysis ran for 10 000 000 generations, the final standard deviation was 0.005589 and the final potential scale reduction factor (PSRF) was 1.000.

Shisa proteins were only found among bilaterian species, although not in all of them. Species such as *Ciona intestinalis*, an urochordate, or *L. gigantea*, a mollusc, have lost this family (table 1). The phylogenies revealed that this family is organized in two subfamilies. The ancestral shisa gene would have appeared in bilaterians and duplicated before their diversification to generate these two subfamilies (electronic supplementary material, figure S2C). Based on the proteins they contain, we have termed them Shisa1/L1 and ShisaL2 (figure 4; electronic supplementary material, figure S6). The Shisa1/L1 subfamily is better conserved, as it has only been lost in echinoderms; instead, the ShisaL2 is only found in deuterostome species, having been lost in the ancestor of protostomes. While the Shisa1/L1 greatly expanded in vertebrates, the ShisaL2 expanded in cephalochordates and hemichordates. Protostome species with shisas show a low number of sequences compared to chordates and *S. kowalevskii*, having 1 or 2 genes per species. The ML phylogeny shows how the Shisa1/L1 subfamily might be further divided in two classes: one comprising vertebrate Shisa4 and Shisa5 with invertebrate sequences and a second including vertebrate shisas: L1, 2, 3 and 6 to 9 (electronic supplementary material, figure S6). Nevertheless, the tree constructed with the BI method presents a different topology for the Shisa1/L1 subfamily (figure 4); this does not allow us to fully conclude that there would be two classes within the Shisa1/L1 subfamily, one being specific to vertebrates, as we found for Type I TARPs.

Vertebrate proteins from the Shisa1/L1 perform very different biological functions. Shisa5 is involved in p53-induced apoptosis, Shisa2 in FGF receptor traffic, Shisa3 in WNT receptor function and Shisas 6 to 9 are ARASs. Due to its position in both phylogenies and its grouping with invertebrate shisas, we propose that Shisa5 would be phylogenetically closer to the ancestral shisa than the other paralogues and that the ancestral gene would also be involved in apoptosis. In this scenario the emergence of ARAS in this subfamily would be the result of neofunctionalization events occurring during vertebrate evolution in the branch of Shisa6 to Shisa9.

### 3.5. The Dispanin C family is poorly conserved among metazoans

**Figure 5.**
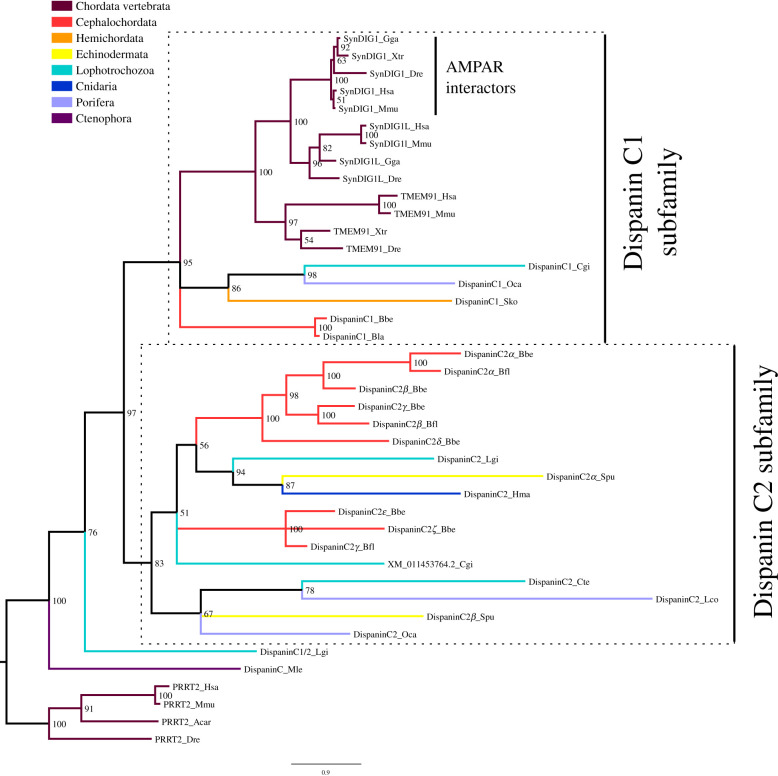
Bayesian inference phylogenetic tree of the Dispanin C family. The two subfamilies in which the Dispanin C family is divided are highlighted by dashed line boxes. The name of each subfamily is presented at the right of the box. Posterior probabilities are shown at tree nodes and protein names at the end of each branch. Tree branches are coloured based on phylum, as indicated in the legend. The vertebrate proteins PRRT2 from the Dispanin B family were used as outgroup. The function of vertebrate sequences, if known, is also indicated. Scale bar denotes number of amino acid substitutions per site. The amino acid substitution model used was Vt + I + G + F, the analysis ran for 10 000 000 generations, the final standard deviation was 0.003312 and the final potential scale reduction factor (PSRF) was 1.000.

SynDIG1, a member of the Dispanin C family, has recently been reported as an AMPAR auxiliary subunit [75]. Our phylogenies indicate that members of this family can be traced to the early-diverging phylum of Ctenophores (figure 5; electronic supplementary material, figure S7). Yet, this family has been lost in multiple lineages and species during metazoan evolution (table 1). Only half of the 26 species investigated present at least one member of the Dispanin C family. Our trees indicate that in metazoans this family is also divided into two subfamilies, which we have named Dispanin C1 and Dispanin C2. The *L. gigantea* DispaninC1/2_Lgi does not belong to any subfamily, since we didn't find other proteins in the same tree branch we did not define a new subfamily for it. The ctenophore sequence DispaninC_Mle is the first to diverge, not belonging to any subfamily; thus we propose that the ancestor of all metazoans had a single gene coding for Dispanin C and after the split of ctenophores it duplicated, giving rise to the two subfamilies (electronic supplementary material, figure S2D). Virtually nothing is known about the two vertebrate paralogues of SynDIG1, SynDIG1L and TMEM91, although SynDIG1L has been found downregulated in mouse models of Huntington's disease [57]. Due to the lack of functional information in other vertebrate or invertebrate Dispanin Cs it not possible to establish when SynDIG1 function as ARAS arose during evolution.

## 4. Discussion

Evolutionary studies have demonstrated that the synaptic proteome has importantly increased in vertebrates [76], expanding the molecular tools available to synaptic physiology [77–79] and multiplying synaptic molecular types [80,81]. The two rounds of whole-genome duplication (2R) that occurred at the base of the vertebrate lineage [82] were the major driving force behind this expansion, as genes expressed at the synapse were retained at high frequencies after these duplication events [83]. The result of this increased complexity in the vertebrate synaptic proteome has been associated with the higher cognitive functions found in mammals [84]. Nevertheless, there are exceptions to this general model of synaptic proteome evolution. This is the case of glutamate receptors, key nervous system proteins driving the excitatory synaptic transmission. These proteins have undergone a highly sophisticated evolutionary pattern in animals, with many lineage-specific gains, losses and expansions of entire classes of these receptors. This process has resulted in a similar number of glutamate receptors being present in most animal species, regardless of the complexity of their nervous system [2]. Here, we present how the neo/subfunctionalization of unrelated proteins into ARASs has resulted in a different evolutionary strategy to increase the synaptic proteome in vertebrates.

Mammalian ARASs were thought to belong to five protein families: CACNG, GSG1, cornichon, sisha and Dispanin C. CACNGs and GSG1s are known to belong to the superfamily of claudins [85], a large group of proteins that presents over 40 members in mammals and that includes three families, one of them being that of CACNGs [86]. Nevertheless, the exact position of GSG1s within claudins was unknown. Our phylogenetic analysis indicates that GSG1s actually belong to the CACNG family of claudins. We thus refer to this family as CACNG-GSG1. Therefore, ARASs are organized into four evolutionarily unrelated protein families. In contrast with what we reported for the family of ionotropic glutamate receptors, which diversified into 12 phylogenetic groups, including four subfamilies and ten classes [2], the animal evolution of the families containing ARAS has not been particularly complex. cornichon, sisha and Dispanin C families can be divided into just two phylogenetic groups (subfamilies) and the CACNG-GSG1 family in four. Nevertheless, all these families have increased their members along animal evolution, and vertebrates have especially increased their number of ARAS. This is particularly true for the CACNG-GSG1 and shisa families, which include 11 and 10 proteins, respectively, in the vertebrate species investigated, of which 7 and 4 are ARASs. Altogether vertebrates generally present 14 ARASs, while, based on our study, invertebrate bilaterals would have fewer, between 1 and 7, and non-bilaterals even fewer, 1 or 2. Sequences from invertebrate bilaterals only fall confidently in two subfamilies with experimentally identified ARAS: that of TARPs within the CACNG-GSG1 family, and that of CNIH1/2/3 among cornichons. Furthermore, non-bilaterals would only present proteins phylogenetically related to the ARAS subfamily of CNIH1/2/3 cornichons. Additionally, a number of invertebrates, including the basal sponge *O. carmela*, could have retained one Dispanin C1 phylogenetically close to the mammalian ARAS SynDIG1, although the phylogenies are not fully conclusive in this regard. The loss of the shisa and Dispanin C families in multiple invertebrate species, and even in entire phyla, suggests a less relevant role of these proteins in invertebrates as compared with vertebrates, which present high conservation levels in both families. Furthermore, our analysis suggests that ARAS from the shisa family are only present in vertebrates, postulating them as an innovation of this lineage.

As ARASs belong to four unrelated protein families that include proteins with other functions, we aimed at using our phylogenetic study to propose when in evolution these proteins acquired their function as AMPAR modulators, although functional information would be required to completely establish their role. Our data indicate that all proteins identified in the TARP subfamily might function as ARASs, which would mean that the TARP subfamily would be the only one in which their proteins are solely dedicated to modulating AMPAR function. On the other hand, we found a possible example of vertebrate neofunctionalization of ARASs in the subfamily of GSG1Ls, as its vertebrate paralogue GSG1 is involved in trafficking of TPAP. Identifying the function of non-vertebrate GSG1s would be required to establish more conclusively this event of neofunctionalization. Vertebrates have two cornichons acting as ARASs (CNIH2 and 3), emerging from a vertebrate-specific duplication. Their closest paralogue is CNIH1, which is involved in TGF*α* maturation. Interestingly, the fly sole orthologue to CNIH1, 2 and 3 is known to participate in the maturation of TGF*α* [18], while the unique orthologue of these proteins in *C. elegans* has a well-proven role as an ARAS [16]. We thus propose that invertebrate orthologues of CNIH1/2/3 perform both functions and that vertebrate paralogues underwent a process of subfunctionalization by which CNIH1 retained the TGF*α* maturation function and the ancestor of CNIH2 and 3 retained the ARAS role. Within the shisa family all known ARASs (Shisa6 to 9) are in the Shisa1/L1 subfamily, yet this phylogenetic group also contains proteins performing other functions, as Shisa5 participates in p53-induced apoptosis [49] and Shisa2 and 3, the closest paralogues to Shisas6–9 with known function, are involved in FGF [47] and WNT [48] receptor trafficking, respectively. In opposition to what we found in the CACNG-GSG1 and cornichon subfamilies containing ARAS, the Shisa1/L1 subfamily includes very few sequences from non-vertebrates. Thereby, TARPs and CNIH1/2/3 subfamilies present 31 and 23 sequences from invertebrate species, respectively, while the Shisa1/L1 subfamily includes only eight. Importantly, all these eight sequences fall in the branch of Shisa5, which is not an ARAS. Thus, due to the topology of the phylogenetic trees we propose that Shisa6 to 9 would have experienced a process of neo/subfunctionalization early in the vertebrate lineage to become ARASs. Finally, the phylogenetic evolution of the Dispanin C family would also suggest a neo/subfunctionalization process in the vertebrate lineage resulting in members of the C1 subfamily becoming ARASs, although, as before, research on vertebrate and invertebrate orthologues of SynDIG1 will be required to fully validate this hypothesis. The fact that large-scale proteomics experiments of synaptic preparations [77–79] and repositories of synaptic proteins [87] do not identify SynDIG1L or TMEM91 suggests that they are unlikely to function as ARASs, thus supporting the hypothesis of a neo/subfunctionalization process of ARASs in this family.

In summary, this study reveals that the large set of ARASs found in vertebrates is absent from other species. Interestingly, the surge in ARASs happened much later than the emergence of AMPARs [2]. The class of AMPA receptors appears and diversifies in the ancestor of bilateral species, around 800 Ma, while the increase in ARAS occurs early in vertebrate evolution, approximately 400 Ma [11]. Our analysis suggests that this increase is due to an expansion of proteins belonging to the TARP subfamily, the neofunctionalization of a group of Shisa1/L1 subfamily proteins and parallel processes of neo/subfunctionalization occurring in the cornichon and Dispanin C families. All these resulted in the recruitment of a large number of ARASs early in vertebrate evolution, which suggests that these proteins might have importantly contributed to the development of the complex nervous systems found in these animals. The parallel recruitment of unrelated proteins to perform synaptic functions represents another strategy by which evolution has favoured an increased complexity in the synaptic proteome.

## Supplementary Material

Supplementary Figures

## Supplementary Material

Table S1

## Supplementary Material

File S1

## Supplementary Material

File S2

## Supplementary Material

File S3

## Supplementary Material

File S4

## Data accessibility

This article has no additional data.
