# Correction to ‘AMPA receptor auxiliary subunits emerged during early vertebrate evolution by neo/subfunctionalization of unrelated proteins’

**DOI:** 10.1098/rsob.200374

**Published:** 2020-12-16

**Authors:** D. Ramos-Vicente, À. Bayés

*Open Biol.* **10**, 200234 (Published Online 28 October 2020) (doi:10.1098/rsob.200234)

