# Supplementary material for: AMPA receptor auxiliary subunits emerged during early vertebrate evolution by neo/subfunctionalization of unrelated proteins
Source: Open Biol. 2020 Oct 28;10(10):200234. doi: 10.1098/rsob.200234 (PMC7653359; doi:10.1098/rsob.200234)
Supplement: Supplementary Figures [file rsob200234supp1.pdf]

AMPA Receptor Auxiliary Subunits Emerged During Vertebrate Evolution by Neo/Subfunctionalization of Unrelated Proteins

David Ramos-Vicente[1,2] and Àlex Bayés[1,2*]

1. Molecular Physiology of the Synapse Laboratory, Biomedical Research Institute Sant Pau, Barcelona, Spain.
2. Universitat Autònoma de Barcelona, Barcelona, Spain.

* Corresponding author
Àlex Bayés, abayesp@santpau.cat
Molecular Physiology of the Synapse Laboratory
IIB Sant Pau
C/Sant Quintí, 77-79
08041 Barcelona, Spain

SUPPLEMENTARY MATERIAL - FIGURES

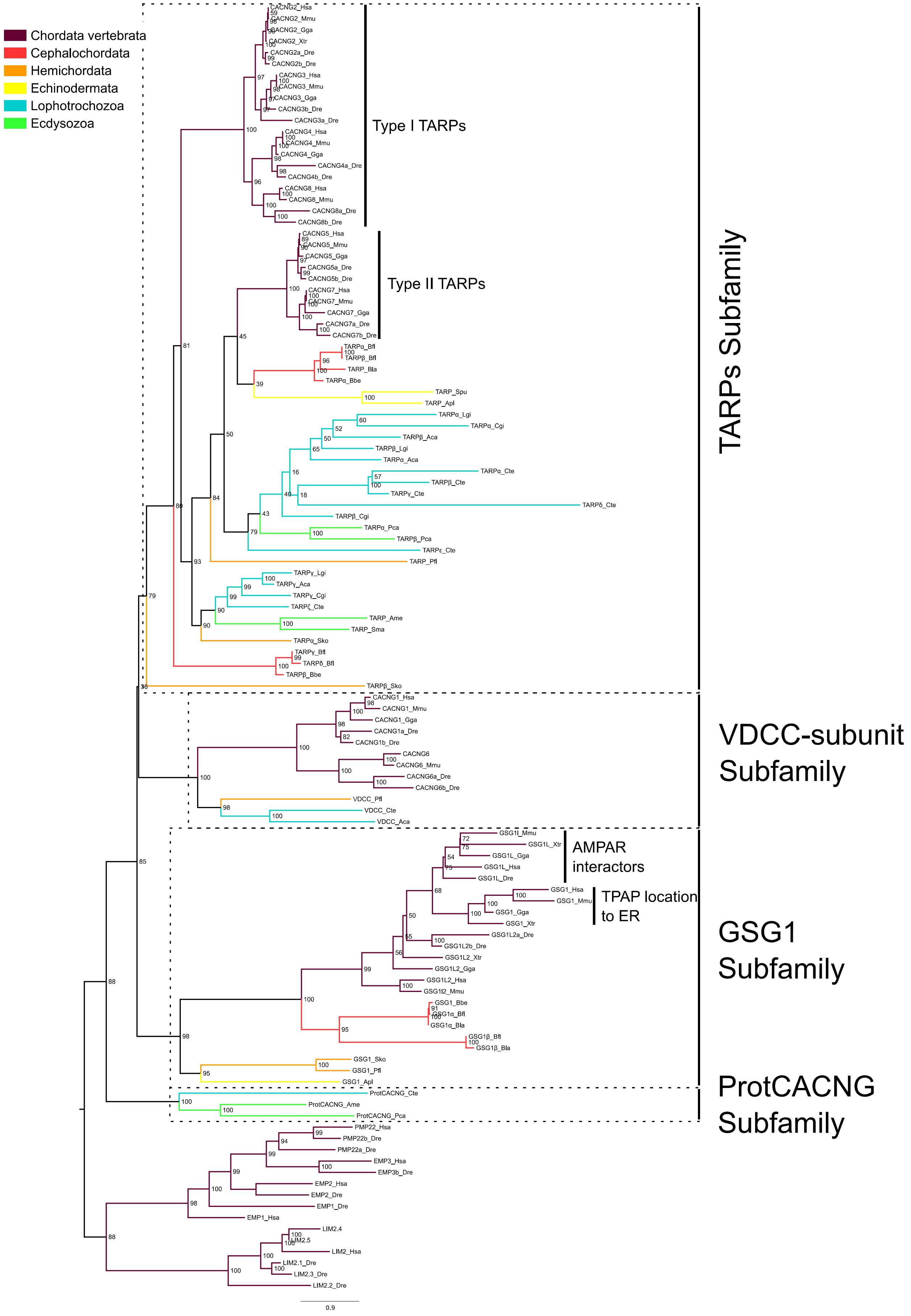

Supplementary Figure 1. Maximum likelihood phylogenetic tree of CACNG-GSG1 protein family.

The subfamilies in which the CACNG-GSG1 protein family is divided are highlighted by dashed line boxes. The name of each subfamily is presented at the right of the box. Bootstrap values are shown at tree nodes and protein names at the end of each branch. Tree branches are coloured based on phylum, as indicated in the legend. The closest relatives to vertebrate CACNG-GSG1 in the Claudin superfamily were used as outgroup. If known, the function of vertebrate GSG1 subfamily sequences was marked on the right of sequence name. Scale bar denotes number of amino acid substitutions per site. The amino-acid substitution model used was Vt+G+F, branch support was obtained after 1000 iterations of ultrafast bootstrapping.

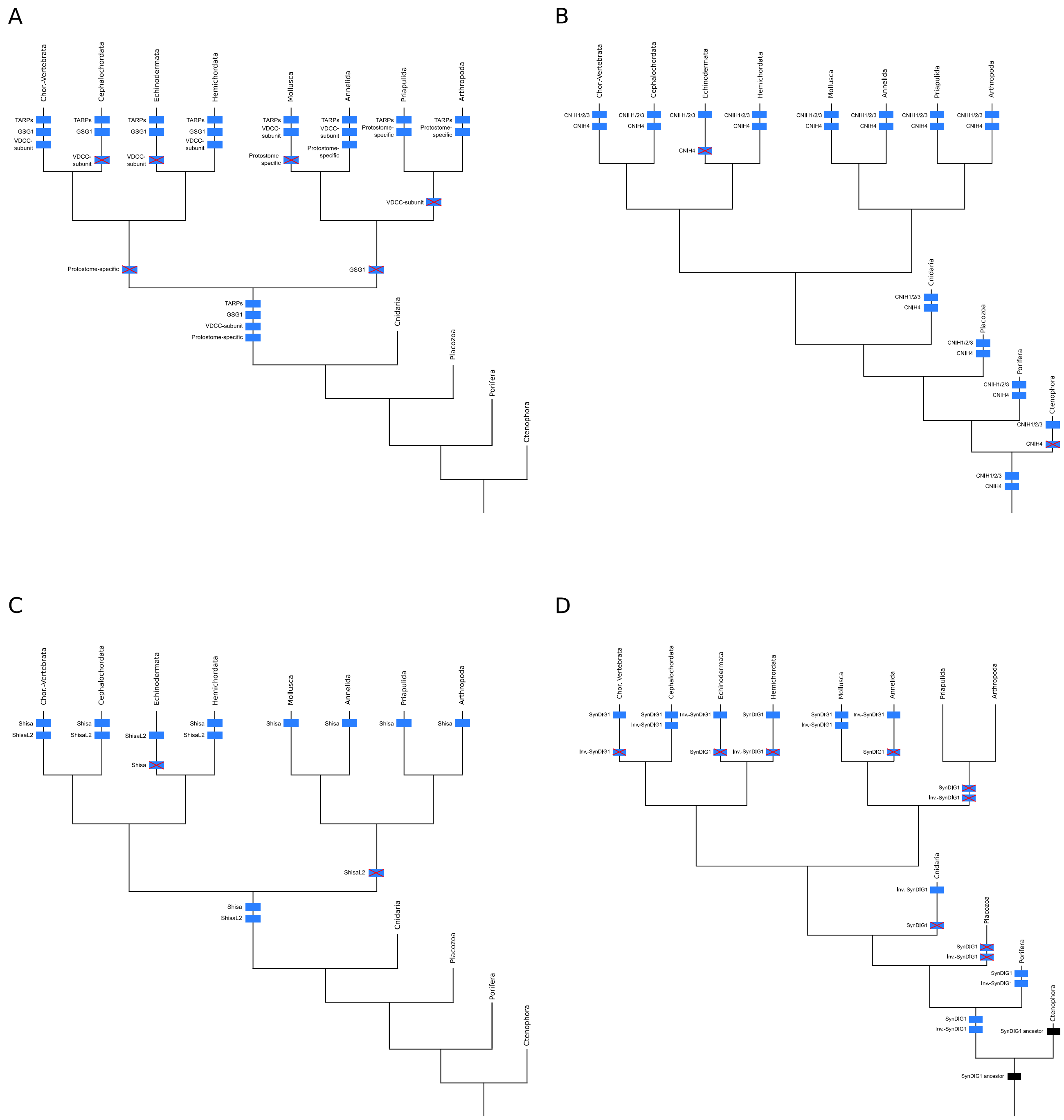

Supplementary Figure 2. Schemes of the evolution of the three protein families and the Dispanin C subfamily.

Schematic trees showing the evolution of (A) CACNG-GSG1, (B) Cornichon, (C) Sisha and (D) Dispanin C families. Each branch corresponds with one lineage. Phylogenetic subfamilies, and classes in the case of Dispanin C tree, are represented by blue boxes. The ancestral SynDIG1 gene is represented by a black box. When a subfamily or class is lost in a lineage or in an ancestor, the corresponding box is crossed out by a red cross. Mollusca and annelida are lophotrochozoans and priapulida and arthropoda ecdysozoans.

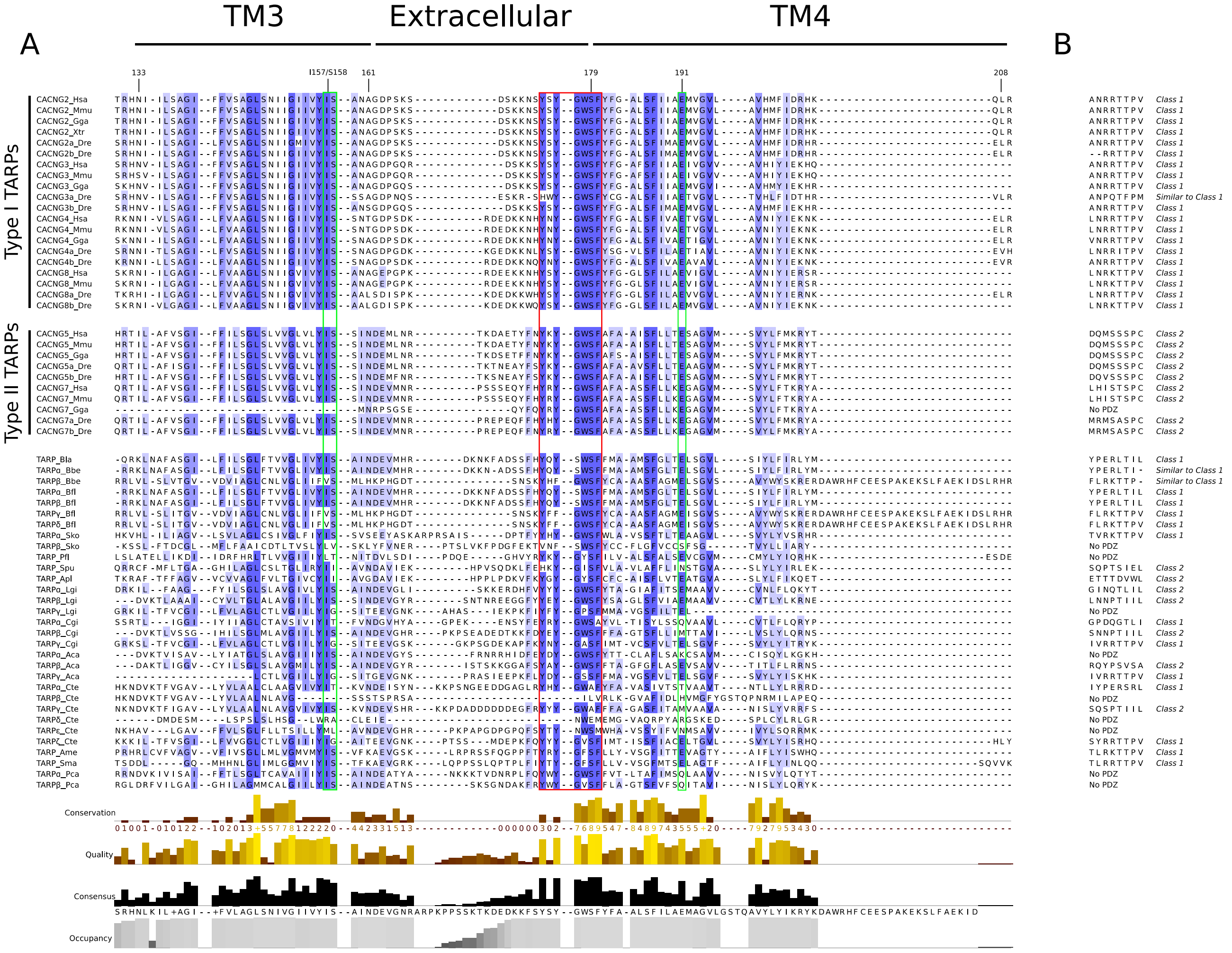

Supplementary Figure 3. Multiple sequence alignment of the TARP subfamily.

Sequence alignment presenting sequences from the TARP subfamily of CACNG-GSG1s. A. Alignment of the TM3 and TM4, which are implicated in the interaction with AMPAR. The extension of the transmembrane segments and the extracellular loop is marked on the top of the alignment. The conservation of each position of the alignment is represented by an intensity gradient of the background, higher conservation corresponding to more intense blue, and by a bar chart at the bottom. Also the quality chart (a measure of the probability of seeing mutation in an alignment position), the consensus sequence and the occupancy chart are shown in the bottom. Protein numbering corresponds to human CACNG2 sequence. B. The last eight residues of proteins containing a PDZ binding motif are shown. If no PDZ binding motif is found, it is labelled as No PDZ. The class of each PDZ binding motif is shown at the left of the amino-acid sequence. Figure was prepared with Jalview v2.11.0.

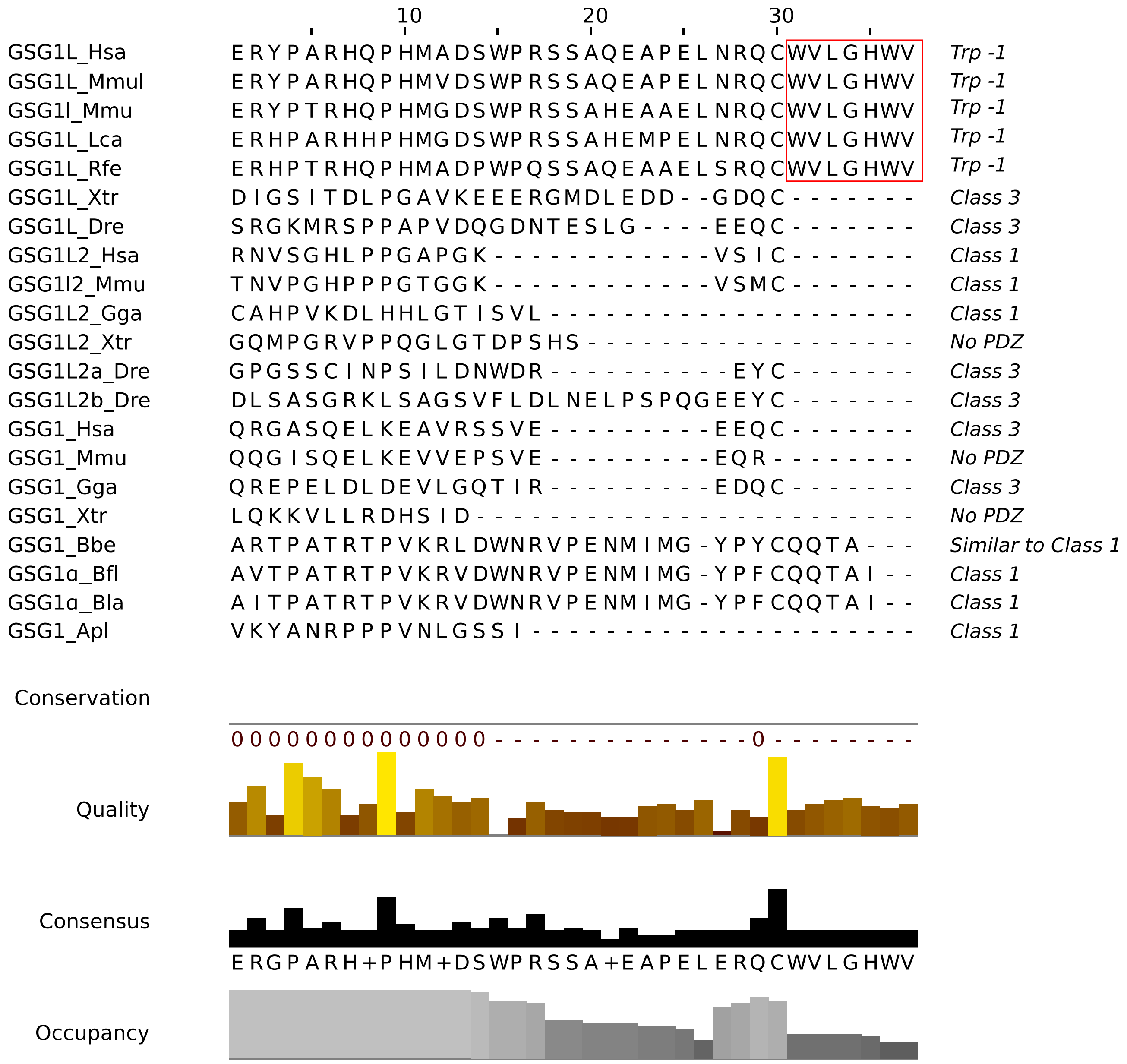

Supplementary Figure 4. Multiple sequence alignment of the C-terminal amino acids of members of the GSG1 subfamily.

We used the software ELM to predict the class of PDZ binding motifs present at each C-terminus of GSG1s, if any. These are indicated. Note the insertion of 7 residues (WVLGHWV) found at the end of mammalian sequences only and highlighted by a red frame. Mammalian species are: *Homo sapiens* (Hsa), *Mus musculus* (Mus); *Macaca mulatta* (Mmul), *Xenopus tropicalis* (Xtr), *Lynx canadensis* (Lca) and Rhinolophus ferrumequinum, bat (Rfe). Figure was prepared with Jalview v2.11.0.

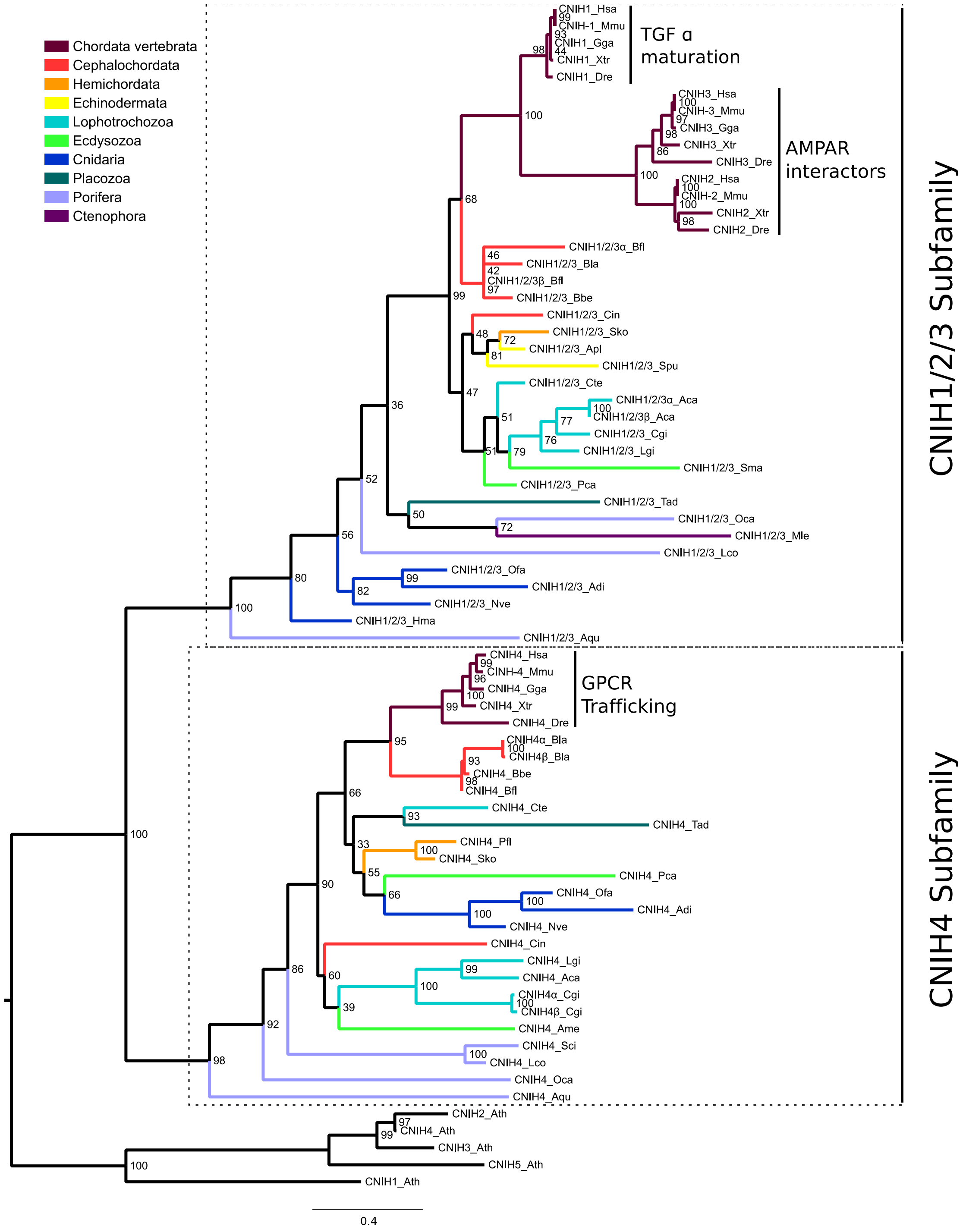

Supplementary Figure 5. Maximum likelihood phylogenetic tree of Cornichon protein family. The two subfamilies in which the Cornichon family is divided are highlighted by dashed line boxes. The name of each subfamily is presented at the right of the box. Bootstrap values are shown at tree nodes and protein names at the end of each branch. Tree branches are coloured based on phylum, as indicated in the legend. Cornichon proteins from *Arabidopsis thaliana* were used as outgroup. The function of vertebrate sequences is indicated. Scale bar denotes number of amino acid substitutions per site. The amino-acid substitution model used was Lg+G, branch support was obtained after 1000 iterations of ultrafast bootstrapping.

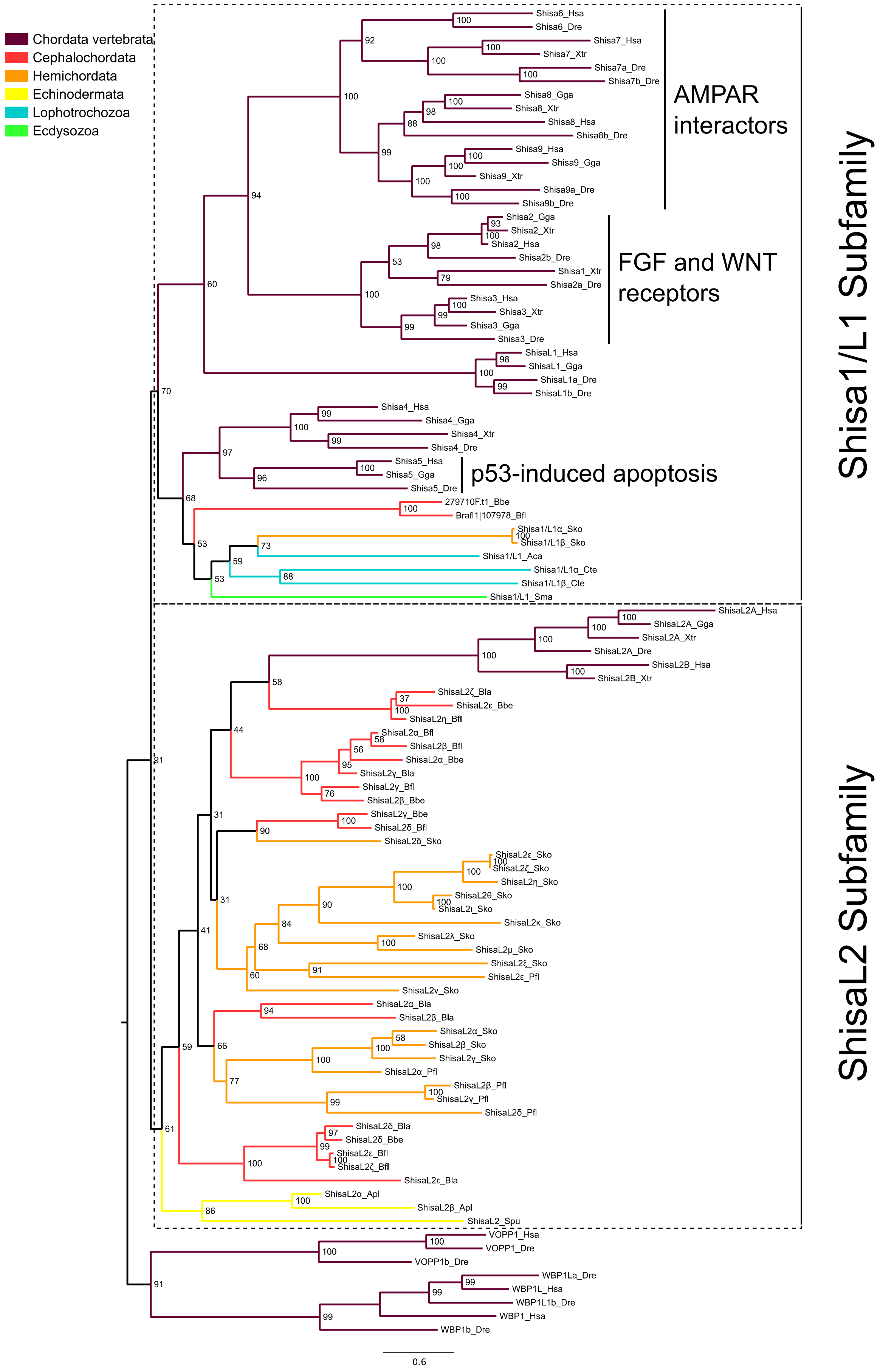
Chordata vertebrata
Cephalochordata
Hemichordata
Echinodermata
Lophotrochozoa
Ecdysozoa
AMPAR interactors
FGF and WNT receptors
p53-induced apoptosis
Shisa1/L1 Subfamily
ShisaL2 Subfamily
0.6

Supplementary Figure 6. Maximum likelihood phylogenetic tree of Shisa protein family.

The two subfamilies in which the Shisa family is divided are highlighted by dashed line boxes. The name of each subfamily is presented at the right of the box. Posterior probabilities are shown at tree nodes and protein names at the end of each branch. Tree branches are coloured based on phylum, as indicated in the legend. The closely related vertebrate proteins VOPP and WBP1 were used as the outgroup. The function of vertebrate sequences, if known, is indicatede. Scale bar denotes number of amino acid substitutions per site. The amino-acid substitution model used was Vt+I+G+F, branch support was obtained after 1000 iterations of ultrafast bootstrapping.

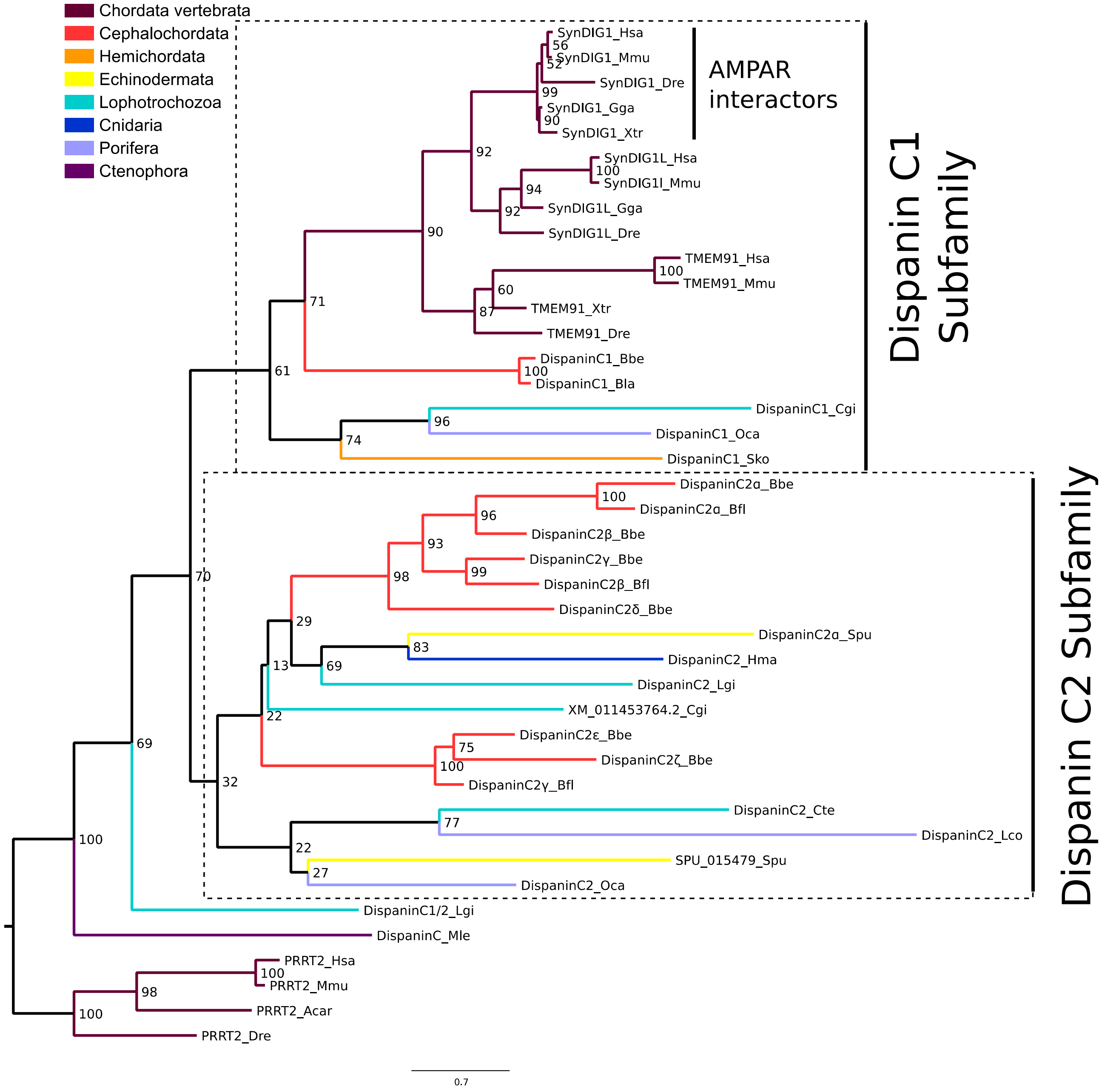

Supplementary Figure 7. Maximum likelihood phylogenetic tree of Dispanin C subfamily. The two subfamilies in which the Dispanin C family is divided are highlighted by dashed line boxes. The name of each subfamily is presented at the right of the corresponding box. Posterior probabilities are shown at tree nodes and protein names at the end of each branch. Tree branches are coloured based on phylum, as indicated in the legend. The vertebrate proteins PRRT2 from the Dispanin B family were used as outgroup. The function of vertebrate sequences, if known, is indicated. Scale bar denotes number of amino acid substitutions per site. The amino-acid substitution model used was Vt+I+G+F, branch support was obtained after 1000 iterations of ultrafast bootstrapping.

OTHER SUPPLEMENATRY FILES

Supplementary Table 1. Reference Table for Protein names and Gene reference codes.

Supplementary File 1. Multiple sequence alignment of CACNG-GSG1 protein family.

Supplementary File 2. Multiple sequence alignment of Cornichon protein family.

Supplementary File 3. Multiple sequence alignment of Shisa protein family.

Supplementary File 4. Multiple sequence alignment of Dispanin C protein family.
